# Development of a Physical Activity Triggers Questionnaire

**DOI:** 10.3390/healthcare11010025

**Published:** 2022-12-22

**Authors:** Yunbo Wang, Hyoung-Kil Kang

**Affiliations:** 1 School of Physical Education, Changchun Normal University, Changchun 130032, China; 2 Department of Physical Education, Kyungnam University, Changwon 51767, Republic of Korea

**Keywords:** college students, physical activity, triggers, scale development

## Abstract

Background: This study aimed to develop a Physical Activity Triggers Questionnaire for Chinese college students and to evaluate its reliability and validity. Methods: On the theoretical basis of the Fogg behavior model and semi-open interviews, an initial questionnaire with 18 items was compiled. The initial questionnaire was administered to 575 students, and to examine its reliability and validity, item discrimination analysis, correlation analysis, homogeneity test, and exploratory factor analysis were conducted using SPSS 26.0. After the examination of the initial questionnaire, the initial 18 items were reduced to 14. The 14-item questionnaire was administered to 621 college students, and with the data, correlation analysis, confirmatory factor analysis, validity test, and reliability test were conducted. Results: To examine the psychometric properties of the 18 items, exploratory factor analysis and confirmatory factor analysis were conducted, and their reliability and validity were examined. After the first round of item development analysis, four items were removed, and a triggers questionnaire with 14 items was developed. The 14 items had three dimensions, including spark, signal, and facilitator triggers, and the cumulative explained variance of the three dimensions was 61.21%. The confirmatory factor analysis of the three dimensions of the 14 items indicated appropriate scale fit indices. The internal consistency reliability, split-half reliability, and test–retest reliability of the 14 items were 0.925, 0.821, and 0.860, respectively, showing that the items have appropriate reliability. Conclusions: The Physical Activity Triggers Questionnaire of the study has acceptable reliability and validity. It is the first questionnaire to measure Chinese college students’ triggers of physical activity and will provide a new basis for the understanding of psychometric properties of physical activity triggers. In addition, the future findings collected from the developed triggers questionnaire can be used to develop strategies to promote health among college students.

## 1. Introduction

Regular physical activity produces social, psychological, and physical benefits for teenagers [1]. The World Health Organization reported that “In adolescents, physical activity confers benefits for the following health outcomes such as improved physical fitness, cardiometabolic health, bone health, cognitive outcomes, mental health, and reduced adiposity” [2] (p. 1). Despite that, approximately two million deaths per year are related to physical inactivity, and a sedentary lifestyle is one of the ten leading causes of disability and death in the world [3]. Lack of physical activity has become a worldwide public health and social problem [4]. This global trend is no exception for Chinese college students. Fitness levels among college students in China have continued to decline since 2010, while obesity rates have steadily increased [5]. This downward trend in physical fitness levels has recently accelerated because Chinese college students’ leisure time is more occupied by online games and electronic devices, and at least four hours of their daily leisure time are currently sedentary. As a result, their leisure time physical activity is lower compared to other generational cohorts [6]. In considering the evidenced benefits of physical activity and the increase in the sedentary lifestyle among Chinese college students, there is a growing interest in promoting physical activity among Chinese college students, and this study aims to develop a questionnaire to measure the triggers of the Fogg Behavior Model (FBM) for Chinese college students.

Triggers come from FBM. FBM is a popular behavioral design theory emerging in recent years that can analyze and explain how human behavior changes. FBM was initially proposed by Fogg to guide user behavior. Later, because of its simple and practical characteristics, it was employed in a variety of industries including product promotion, Internet media, and personal management and development [7]. FBM allows a systematic way to examine specific behavior changes through types of motivation and ability as well as strategies or methods for triggering targeted behaviors [8]. Fogg asserted that “The FBM is a new way to understand the drivers of human behavior, and this psychological model identifies and defines three factors that control whether a behavior is performed” [8] (p. 1). The three factors are motivation, ability, and triggers. Fogg stated that “for a person to perform a target behavior, he or she must be sufficiently motivated, have the ability to perform the behavior, and be triggered to perform the behavior” [8] (p. 1). It is above all the triggers that differentiate FBM from other behavioral theories.

Triggers are a visible indicator connected with a predicted change in behavior, which serves as a reminder to people engaging in the target behavior [9,10]. Even if both ability and motivation are sufficient to carry out the activity, it cannot occur in the absence of proper triggers, and expected target behaviors do not happen if the appropriate triggers are not set at the right time [8]. Fogg explained that “A trigger can take many forms—an alarm that sounds, a text message, and so on. Whatever the form, successful triggers have three characteristics: First, we notice the trigger. Second, we associate the trigger with a target behavior. Third, the trigger happens when we are both motivated and able to perform the behavior” [8] (p. 3). As such, triggers play a vital role in initiating behaviors. For example, college students have more or less motivation and ability to perform physical activities. If there is a trigger to remind them, it inevitably increases the success rate of their participation in physical activity.

Fogg referred to the three characteristics of triggers as a spark, facilitator, and signal [8]. Spark is related to motivational elements. A spark can be a type of extrinsic motivation, which can prompt and attract people to perform a target behavior. An inspiring text or a video that encourages target behaviors might be the spark. A facilitator is more suitable for individuals who do not have sufficient ability but have clear motivations. A facilitator has clear guidance on behavior, which makes it easier to implement the behavior. A signal serves as a reminder when people have both the capacity and willingness to conduct the goal activity, but it does not serve as a motivator [11]. Previous research addressing FBM has only used interviews or controlled experiment designs to empirically examine triggers because there is no developed triggers questionnaire [12,13,14]. In response to the lack of tools to measure triggers, this study is the first to develop a triggers questionnaire for accessing Chinese college students’ physical activity through semi-open interviews with college students and expert interviews [15,16]. This questionnaire provides a new quantitative measure of Chinese college students’ physical activity behavior and provides foundational information for the follow-up triggers research on college students’ physical activity. In addition, this study will extend the scope of FBM research and help to identify interventions aimed at promoting physical activity among Chinese college students

## 2. Materials and Methods

Two-hour semi-open interviews were conducted with 27 college students to develop initial triggers items. The interview questions were “Do you engage in physical activity in your leisure time?”, “What is your reason you do not engage in physical activity in your leisure time?”, and “What do factors trigger your physical activity? Please give examples”. After the semi-open interviews, a total of 22 initial trigger items were developed with the three dimensions of spark, facilitator, and signal.

The face validity of the 22 items was examined by two psychology professors and four physical education teachers, and four of the 22 items were removed. With the 18 items, a pilot study was conducted with 32 college students, and the students reported that the content of the questionnaire was concise and easy to understand, and potentially covered all possible physical activity triggers that they could think of. The 18 items were measured on a five-point Likert scale (1, strongly disagree–5, strongly agree) and consisted of six items for the spark dimension, five items for the signal dimension, and seven items for the facilitator dimension. The 18-item questionnaire was administered to 575 students, and with the data, item discriminant analysis, correlation analysis, homogeneity test, and exploratory factor analysis were conducted to further refine the items in each dimension of the scale and examine the psychological structure of the items. After the examination of the 18-item questionnaire, 621 college students were randomly selected to participate in the formal scale measurement. Correlation analysis, confirmatory factor analysis, validity test, and reliability test were conducted for the formal questionnaire. AMOS 24.0 statistical software was used to conduct confirmatory factor analysis on the structure of the triggers items, and model fit indexes were used to examine the triggers constructions. SPSS 26.0 statistical software was used to analyze the reliability of the triggers items.

## 3. Results

In 2021, 575 college students from Changchun Normal University participated in this study to examine the psychometric properties of the 18 items. After excluding 32 incomplete questionnaires, 543 questionnaires were used for the data analysis.

### 3.1. Item Discrimination Analysis

SPSS 26.0 statistical software was used to analyze the 18 items with the three dimensions of trigger factors. Based on Kelley’s derivation [17], this study divided participants into high and low groups, with the top 27% of the total scores being the high group and the bottom 27% comprising the low group. Between the high and the low groups, independent sample t-tests were conducted to examine item discrimination. The results showed that all items reached a significance level (*p* < 0.001) of mean differences between the high and low groups, indicating acceptable item discrimination, presented in Table 1.

### 3.2. Correlation Analysis

The correlation coefficient between the total score and each of the 18 items is more than 0.5, and all the correlation coefficients reached a significance level at *p* < 0.001, indicating each item reflects the contents of a psychological construct, presented in Table 2 [18].

### 3.3. Homogeneity Test

To examine the homogeneity of the 18 items, this study conducted Cronbach’s alpha test, and the alpha coefficient of the overall scale was 0.919, indicating that the internal consistency of the 18 items was appropriate [19]. In addition, all the items’ corrected item total correlations were between 0.498 and 0.691, indicating that the homogeneity of the 18 items is acceptable, as presented in Table 3 [18].

### 3.4. Exploratory Factor Analysis (EFA)

The EFA of the 18 developed items was conducted, and the KMO value of the items was 0.937, indicating that the items are appropriate for EFA, as presented in Table 4 [20].

After the EFA was conducted, item 12 was deleted because it had a factor loading lower than 0.4, and items 11, 13, and 14 were deleted because they showed cross-factor loadings. As a result, a total of four items were deleted, and 14 items remained. The results of EFA are presented in Table 5.

### 3.5. Name of the Factors

Based on the meanings of the items and the conceptual basis of FBM, three factors were named, and presented in Table 6.

### 3.6. Analysis of the Developed 14 Items

#### 3.6.1. Demographic Characteristics of the Subjects

Using a random sampling method, 621 college students from Changchun Normal University participated in this study. After 25 incomplete questionnaires were excluded, 596 questionnaires were used for data analysis. The participants’ demographic information is presented in Table 7.

#### 3.6.2. Total Correlation between the Total Score and Each Item

If the correlation between the total score and each item is less than 0.4, the item is not suitable for reflecting the content to be measured [19]. The correlation coefficient between the total score and each of the items is more than 0.6, and all the correlation coefficients reached a significance level at *p* < 0.001, indicating that each item reflects the content to be purposively measured, as presented in Table 8.

### 3.7. Confirmatory Factor Analysis (CFA)

**Figure 1:**
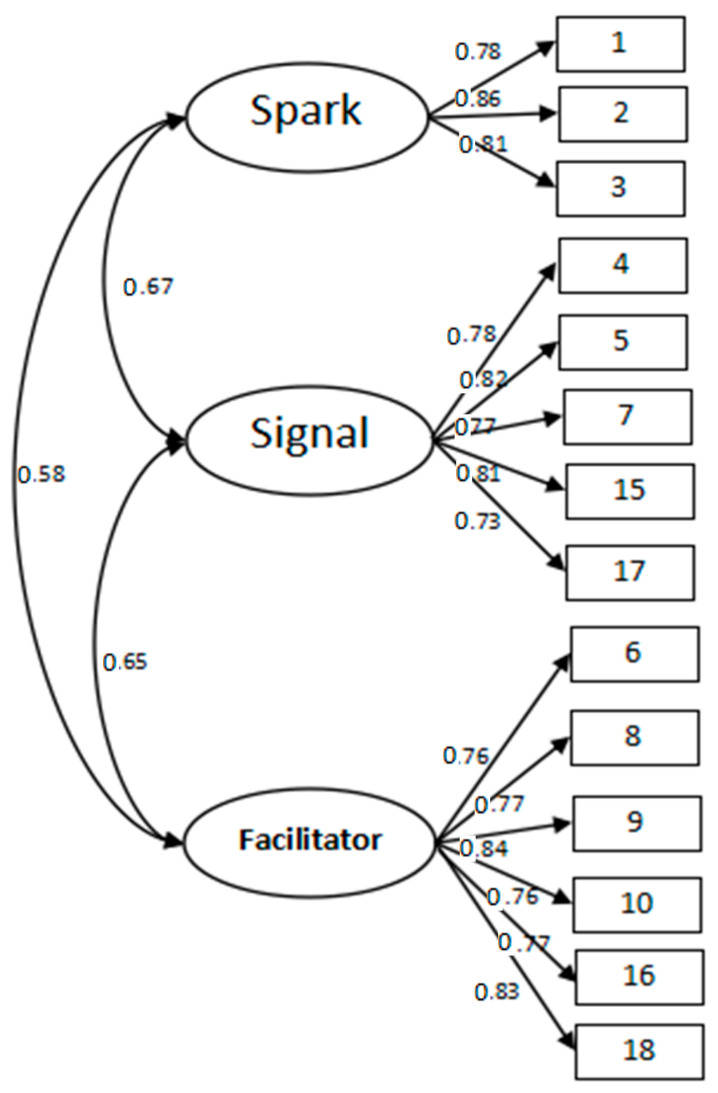
Confirmatory Factor Analysis of the 14 items.

Using Amos 24.0, CFA was conducted with the three latent variables including signal, facilitator, and spark. The latent variables of the signal, facilitator, and spark had five (5, 17, 4, 15, and 7), six (9, 16, 8, 18, 10, and 6), and three (1, 2, and 3) observed variables, respectively, presented in Figure 1.

In Table 9, the CFA showed that x^2^/df is 2.476 (<3) [21,22,23], RMSEA is 0.050 (<0.05), NFI, IFI, CFI, and TLI are all above 0.9 [24,25], indicating appropriate fitting indexes.

### 3.8. Validity Test

#### 3.8.1. Convergent Validity

In Table 10, the factor loadings of signal, facilitator, and spark corresponding to each item are all greater than 0.6. In addition, all the AVE values and CR values are over 0.5 and 0.8, respectively, indicating that the convergent validity of the 14 items is acceptable [26].

#### 3.8.2. Discriminant Validity

The discriminant validity can be evaluated by using the Fornell–Lacker criterion [26]. This method compares the square root of the average variance extracted (AVE) with the correlation of latent constructs. It can be seen from Table 11 that there is a significant correlation between signal, facilitator, and spark (*p* < 0.01), and the square root of AVE is greater than the correlation between them. This indicates that all the latent variables in this study are both conceptually and empirically distinct from each other [26,27]. The discriminant validity of the scale is acceptable.

### 3.9. Reliability Test

The reliability of the 14 items was tested using split-half reliability, internal consistency reliability, and test–retest reliability, presented in Table 12. The internal consistency reliability and split-half reliability of the overall scale are 0.925 and 0.821, respectively, indicating that the internal consistency reliability and split-half reliability of the 14 items are acceptable [18].

Three hundred thirty college students from Changchun Normal University were selected to examine the test–retest reliability of the 14 items, and 311 valid questionnaires were used for the analysis. Questionnaires for the first survey were collected on 6 October 2021, and the questionnaires for the second retest reliability were collected on 2 November 2021. The test–retest correlation coefficients of the two-survey data for the three dimensions were spark (0.737), facilitator (0.765), and signal (0.788), indicating acceptable test–retest reliability [28].

## 4. Discussion

Based on FBM, this study compiled a triggers questionnaire to assess Chinese college students’ physical activity behavior for the first time, and proposes that the triggers have three psychological constructs including spark, facilitator, and signal. The developed triggers questionnaire has a total of 14 items, including three items for spark, five items for signal, and six items for facilitator. This study is the first study showing that the conceptually proposed psychological constructs of triggers are empirically correct. The results of the reliability and validity analysis show that the 14 items and three factors have acceptable reliability and validity and can appropriately measure the triggering factors of physical activity of Chinese college students. To the best of our knowledge, there are very few previous studies addressing trigger factors for physical activity, and no study on a triggers questionnaire has been identified. Thus, this discussion is of an exploratory nature, and what follows are presumably theoretical and practical implications of the triggers questionnaire.

The spark items of this study are “See sports events or sports-related content broadcasted by public media (TV, Internet, etc.).”, “See advertisements, banners, leaflets, etc. promoting sports” and “Receive the exercise push message from the SMS or WeChat official account”. All the spark items are associated with unexpected and indirect messages that stimulate and attract Chinese college students to engage in physical activity. In cognitive evaluation theory, social cues that make people feel controlled decrease their intrinsic motivation [29], and Chinese college students who are exposed to randomly distributed unexpected messages are unlikely to feel controlled. In this sense, the spark items possibly promote Chinese college students’ intrinsic motivation for physical activity. Cognitive evaluation theory also suggests that social cues that are informative to recipients are likely to produce positive effects on intrinsic motivation [29], and this notion needs to be adopted when we design sparks-related strategies to promote physical activity. In addition, people’s behaviors are not easily induced by sparks unless people intend to associate the sparks with their behaviors, and sparks that lead to doing something we do not want to do may irritate us [8], all of which needs to be considered when we implement sparks-related approaches to promote physical activity [11].

The facilitator items of this study are “Seeing public figures you admire doing physical activity”, “My parents invited me to do physical activities together, “My parents urged me to do physical activity”, “Seeing people around me that I respect or like doing physical activity”, “My friends (or classmates) urge me to do physical activities, “The doctor advised me to do physical activity”, and the developed facilitator items can be discussed with social learning theory and a socio-ecological model. In social learning theory, observation-based learning such as modeling can occur when people extract information from observation and decide to engage in behavior that is associated with the observation [30]. This learning process can be heightened when the modeling is performed by someone whom the observers admire and respect. Considering this notion, the developed facilitator items of “Seeing public figures you admire doing physical activity” and “Seeing people around me that I respect or like doing physical activity” can be understood.

In addition, socio-ecological model proposes that five layers of social systems, including the microsystem, mesosystem, exosystem, macrosystem, and chronosystem, influence human development and decisions, and the microsystem that has direct interactions with people has the greatest impact on people’s development and decisions [31]. The developed facilitator items are relevant to parents, friends, and doctors with whom college students have direct contact and can be seen as the microsystem of the socio-ecological model. For all these, the developed facilitator items can successfully reflect the triggers of physical activity of Chinese college students. It also needs to be noted that the facilitator plays a significant role in initiating individuals’ target behaviors when the individuals have high motivation but have a low ability for target behaviors [8]. In this regard, instructive information about physical activity may be more successful than motivational information when we implement facilitator approaches to promote physical activity.

The signal items of this study are “Timed reminder of sports watch or mobile phone”, “Regular reminder of the fitness application”, “The alarm reminder for exercise set by myself”, “Join the WeChat exercise group to remind me of physical activity every day”, “See text or pictures posted in your dorm or home reminding yourself to be physically active”. When the individual motivation and ability are sufficient, a signal can initiate target behaviors [8]. A signal is not a motivating factor but can be a prompt or reminder for a target behavior, and it does not have to cause stress to the individual [11]. The tools of signal items in this study include mobile phones, sports watches, alarm clocks, fitness apps, and WeChat groups, which can broadly cover the common reminder tools used by Chinese college students. However, commonly used reminder tools among college students can be changed with the popularity of mobile applications and the development of technology, and this has to be accounted for in future research. In addition, Fogg stated that signal and facilitator are more effective tools to initiate target behaviors, compared to sparks [8], which is worth considering when we employ triggers of FBM to promote physical activity among college students.

The development of this scale provides a new empirical basis for the composition of physical activity triggers and provides new research opportunities to explore the relationships between triggers and other physical activity-related psychological constructs. This scale can be also applied to experimental research to further examine its validity and reliability and to elaborate on the role of triggers in human behavior decisions. The validation of the triggers questionnaire means that a range of human behavior decisions of college students are subject to intervention from the perspective of triggers, and the specific possible approaches are: (1) Encourage the public media or campus administrators to publicize their desired behaviors with indirect but informative messages to generate an internal drive to promote the occurrence of the behaviors. (2) The roles of members of the microsystem in socio-ecological model need to be emphasized in guiding college students to make positive behavior decisions. (3) Use the reminder function of the latest tools to regularly remind college students to initiate behaviors beneficial for them.

This study is not without its limitations. The subjects of this questionnaire are only first- and second-year students of a university in northeast China. Future studies need to broaden the scope of the research by collecting data in various regions of China and by including diverse populations. Along the same lines, when the questionnaire is applied to populations in other countries, its reliability and validity should be reexamined, and some items may need to be adjusted. The content validity of the items can be improved by correlational studies with physical activity-related psychological constructs such as intra constraints in leisure time physical activity and behavioral intentions in planned action theory. Lastly, because of socio-cultural desirability and recall biases, self-administered questionnaires can be biased, and future research needs to consider observation-based research to strengthen the reliability and validity of the developed trigger items.

## 5. Conclusions

This study compiled a “Chinese College Students’ Physical Activity Triggers Questionnaire” for the first time and examined its reliability and validity. Through exploratory factor analysis and confirmatory factor analysis, three factors and 14 items, including three spark items, six facilitator items, and five signal items, were found to be appropriate for measuring triggering factors of physical activity among Chinese college students. This questionnaire can add a new quantitative method to the literature and can provide underpinning information for subsequent intervention research about college students’ physical activity. This study is exploratory in nature as the first study to develop a questionnaire, yet it opens new opportunities for deepening our understanding of people’s behavior decisions with the consideration of the relationship between motivation, ability, and triggers. Future findings based on data collected using the developed triggers questionnaire can be used by health administrators to develop strategies to promote health among college students.

## Figures and Tables

**Table 1:** Mean Differences between High and Low Groups (*N* = 543).

Items	Group	*N*	*M*	*SD*	*t*	*p*
1: See sports events or sports-related content broadcasted by public media (TV, Internet, etc.).	Low Group	162	3.62	0.912	−13.647	0.000
	High Group	155	4.74	0.495		
2: See advertisements, banners, leaflets, etc. promoting sports.	Low Group	162	3.21	0.915	−16.767	0.000
	High Group	155	4.65	0.578		
3: Receive the exercise push message from the SMS or WeChat official account.	Low Group	162	2.93	0.936	−15.328	0.000
	High Group	155	4.42	0.788		
4: The alarm reminder for exercise set by me.	Low Group	162	2.80	1.051	−15.657	0.000
	High Group	155	4.43	0.790		
5: Timed reminder of sports watch or mobile phone.	Low Group	162	2.65	0.942	−20.651	0.000
	High Group	155	4.54	0.667		
6: The doctor advised me to do physical activity.	Low Group	162	3.65	0.916	−13.814	0.000
	High Group	155	4.77	0.477		
7: See text or pictures posted in your dorm or home reminding yourself to be physically active.	Low Group	162	2.69	0.838	−18.747	0.000
	High Group	155	4.41	0.795		
8: My parents urged me to do physical activity.	Low Group	162	3.30	0.925	−16.650	0.000
	High Group	155	4.70	0.537		
9: Seeing public figures you admire doing physical activity.	Low Group	162	3.44	1.039	−15.282	0.000
	High Group	155	4.81	0.457		
10: My friends (or classmates) urge me to do physical activities.	Low Group	162	3.08	0.856	−17.997	0.000
	High Group	155	4.62	0.647		
11: See fitness and other physical exercise videos played from the media such as TikTok and WeChat video account.	Low Group	162	3.15	0.921	−17.437	0.000
	High Group	155	4.63	0.559		
12: See the WeChat sports step rankings or sports APP rankings.	Low Group	162	3.41	1.079	−13.994	0.000
	High Group	155	4.74	0.533		
13: Obtain information about physical fitness test (such as receiving notification of upcoming physical test).	Low Group	162	3.25	1.017	−15.303	0.000
	High Group	155	4.66	0.573		
14: My friends (or classmates) invite me to participate in physical activities.	Low Group	162	3.33	0.834	−18.319	0.000
	High Group	155	4.72	0.466		
15: Join the WeChat exercise group to remind me of physical activity every day.	Low Group	162	2.61	0.836	−21.390	0.000
	High Group	155	4.50	0.733		
16: My parents invited me to do physical activities together.	Low Group	162	3.30	0.835	−16.613	0.000
	High Group	155	4.64	0.580		
17: Regular reminder of the fitness application APP.	Low Group	162	2.64	0.896	−18.789	0.000
	High Group	155	4.40	0.761		
18: Seeing people around me that I respect or like doing physical activity.	Low Group	162	3.60	0.873	−15.192	0.000
	High Group	155	4.77	0.435		

**Table 2:** Correlations.

	1	2	3	4	5	6	7	8	9	10	11	12	13	14	15	16	17	18	Total Score
1	1																		
2	0.573 **	1																	
3	0.443 **	0.568 **	1																
4	0.320 **	0.347 **	0.402 **	1															
5	0.350 **	0.387 **	0.454 **	0.623 **	1														
6	0.297 **	0.340 **	0.247 **	0.314 **	0.397 **	1													
7	0.286 **	0.368 **	0.430 **	0.461 **	0.584 **	0.336 **	1												
8	0.264 **	0.351 **	0.342 **	0.345 **	0.432 **	0.420 **	0.419 **	1											
9	0.351 **	0.357 **	0.319 **	0.242 **	0.323 **	0.391 **	0.358 **	0.411 **	1										
10	0.242 **	0.314 **	0.314 **	0.395 **	0.476 **	0.416 **	0.504 **	0.556 **	0.473 **	1									
11	0.404 **	0.469 **	0.433 **	0.317 **	0.419 **	0.298 **	0.423 **	0.362 **	0.459 **	0.428 **	1								
12	0.260 **	0.353 **	0.348 **	0.268 **	0.308 **	0.284 **	0.327 **	0.334 **	0.275 **	0.409 **	0.419 **	1							
13	0.310 **	0.380 **	0.420 **	0.356 **	0.399 **	0.293 **	0.336 **	0.362 **	0.303 **	0.379 **	0.459 **	0.328 **	1						
14	0.290 **	0.399 **	0.357 **	0.370 **	0.438 **	0.339 **	0.448 **	0.360 **	0.352 **	0.519 **	0.441 **	0.374 **	0.413 **	1					
15	0.329 **	0.410 **	0.398 **	0.469 **	0.558 **	0.315 **	0.546 **	0.382 **	0.381 **	0.524 **	0.506 **	0.365 **	0.420 **	0.439 **	1				
16	0.283 **	0.325 **	0.351 **	0.298 **	0.405 **	0.433 **	0.435 **	0.559 **	0.417 **	0.456 **	0.358 **	0.335 **	0.328 **	0.436 **	0.443 **	1			
17	0.250 **	0.350 **	0.361 **	0.423 **	0.613 **	0.345 **	0.535 **	0.382 **	0.323 **	0.470 **	0.419 **	0.337 **	0.430 **	0.436 **	0.622 **	0.417 **	1		
18	0.316 **	0.350 **	0.347 **	0.243 **	0.343 **	0.329 **	0.382 **	0.356 **	0.490 **	0.383 **	0.410 **	0.304 **	0.381 **	0.417 **	0.378 **	0.430 **	0.356 **	1	
Total score	0.556 **	0.651 **	0.649 **	0.629 **	0.739 **	0.572 **	0.706 **	0.648 **	0.609 **	0.704 **	0.684 **	0.567 **	0.625 **	0.663 **	0.734 **	0.651 **	0.700 **	0.604 **	1

Note: 1–18 represent the 18 items of the “Physical Activity Triggers Questionnaire”, ** *p* < 0.01.

**Table 3:** The overall statistics of each item of the Physical Activity Triggers Questionnaire.

	Scale Mean If Item Deleted	Scale Variance If Item Deleted	Corrected Item Total Correlation	Cronbach’s Alpha If Item Deleted
1	65.08	118.236	0.498	0.917
2	65.35	115.434	0.598	0.915
3	65.65	114.646	0.591	0.915
4	65.66	114.039	0.564	0.916
5	65.69	111.659	0.691	0.912
6	65.04	118.251	0.517	0.917
7	65.69	113.201	0.656	0.913
8	65.25	115.924	0.597	0.915
9	65.08	116.535	0.552	0.916
10	65.37	114.502	0.658	0.913
11	65.35	115.043	0.636	0.914
12	65.18	116.862	0.502	0.917
13	65.33	115.758	0.567	0.916
14	65.23	116.568	0.617	0.915
15	65.68	111.959	0.686	0.913
16	65.26	116.459	0.602	0.915
17	65.71	112.914	0.647	0.914
18	65.01	118.203	0.555	0.916

**Table 4:** KMO and Bartlett’s Test.

KMO	Bartlett’s Test of Sphericity Approx. Chi-Square	*df*	*Sig.*
0.937	4241.400	153	0.000

**Table 5:** Exploratory Factor Analysis Results.

Items	Signal	Facilitator	Spark
5	0.787	0.228	0.239
17	0.733	0.299	0.098
4	0.726	0.073	0.272
15	0.687	0.325	0.204
7	0.675	0.340	0.174
9	0.078	0.719	0.282
16	0.292	0.695	0.119
8	0.322	0.677	0.095
18	0.132	0.627	0.300
10	0.485	0.624	0.015
6	0.216	0.609	0.167
1	0.118	0.205	0.803
2	0.229	0.241	0.785
3	0.371	0.165	0.677
Eigenvalue	6.191	1.212	1.167
Variance	44.220	8.659	8.332
Cumulated variance	44.220	52.879	61.211
Cronbach’s α	0.856	0.822	0.769
Cronbach’s α = 0.902			
Kaiser–Meyer–Olkin value = 0.919			
Bartlett’s test of sphericity = 3241.681 *df* = 91 *sig.* = 0.000			

**Table 6:** Three factors and 14 items.

Factors	Items
Signal	5: Timed reminder of sports watch or mobile phone.
	17: Regular reminder of the fitness application APP.
	4: The alarm reminder for exercise set by me.
	15: Join the WeChat exercise group to remind me of physical activity every day.
	7: See text or pictures posted in your dorm or home reminding yourself to be physically active.
Facilitator	9: Seeing public figures you admire doing physical activity.
	16: My parents invited me to do physical activities together.
	8: My parents urged me to do physical activity.
	18: Seeing people around me that I respect or like doing physical activity.
	10: My friends (or classmates) urge me to do physical activities.
	6: The doctor advised me to do physical activity.
Spark	1: See sports events or sports-related content broadcasted by public media (TV, Internet, etc.).
	2: See advertisements, banners, leaflets, etc. promoting sports.
	3: Receive the exercise push message from the SMS or WeChat official account.

**Table 7:** Demographic Characteristics of the Test Samples.

Variable	Classification	N
Sex	Male	198
	Female	398
Grade	First-year students	433
	Second-year students	163
Age	17	9
	18	152
	19	276
	20	129
	21	30

**Table 8:** Correlations.

	1	2	3	4	5	7	15	17	6	8	9	10	16	18	Total Score
1	1														
2	0.672 **	1													
3	0.632 **	0.696 **	1												
4	0.307 **	0.375 **	0.363 **	1											
5	0.409 **	0.449 **	0.437 **	0.634 **	1										
7	0.339 **	0.394 **	0.372 **	0.583 **	0.630 **	1									
15	0.308 **	0.371 **	0.339 **	0.639 **	0.659 **	0.642 **	1								
17	0.323 **	0.335 **	0.359 **	0.593 **	0.592 **	0.546 **	0.601 **	1							
6	0.412 **	0.405 **	0.400 **	0.425 **	0.461 **	0.420 **	0.403 **	0.406 **	1						
8	0.434 **	0.492 **	0.444 **	0.415 **	0.447 **	0.430 **	0.475 **	0.400 **	0.557 **	1					
9	0.416 **	0.432 **	0.406 **	0.433 **	0.480 **	0.452 **	0.415 **	0.397 **	0.620 **	0.652 **	1				
10	0.440 **	0.433 **	0.413 **	0.350 **	0.448 **	0.437 **	0.241 **	0.390 **	0.572 **	0.554 **	0.656 **	1			
16	0.410 **	0.428 **	0.394 **	0.351 **	0.425 **	0.389 **	0.366 **	0.396 **	0.592 **	0.598 **	0.657 **	0.568 **	1		
18	0.416 **	0.452 **	0.433 **	0.406 **	0.464 **	0.433 **	0.383 **	0.413 **	0.654 **	0.636 **	0.695 **	0.634 **	0.624 **	1	
Total score	0.651 **	0.696 **	0.672 **	0.693 **	0.758 **	0.711 **	0.691 **	0.680 **	0.730 **	0.754 **	0.768 **	0.711 **	0.713 **	0.760 **	1

Note: ** *p* < 0.01.

**Table 9:** Model Fit Indexes of Confirmatory Factor Analysis.

x^2^/df	RMSEA	IFI	CFI	NFI	TLI	RMR
2.476	0.050	0.978	0.978	0.964	0.973	0.033

**Table 10:** Consolidation Validity Index.

Way			Estimate	AVE	CR
9	<---	Facilitator	0.841	0.620	0.907
16	<---	Facilitator	0.767		
8	<---	Facilitator	0.770		
18	<---	Facilitator	0.829		
10	<---	Facilitator	0.755		
6	<---	Facilitator	0.759		
5	<---	Signal	0.822	0.613	0.888
17	<---	Signal	0.735		
4	<---	Signal	0.777		
15	<---	Signal	0.807		
7	<---	Signal	0.771		
1	<---	Spark	0.783	0.669	0.858
3	<---	Spark	0.810		
2	<---	Spark	0.858		

**Table 11:** Discriminant Validity of the Latent Variables.

	Signal	Facilitator	Spark
Signal	0.613		
Facilitator	0.503 **	0.620	
Spark	0.391 **	0.444 **	0.669
Square root of AVE	0.783	0.787	0.818

Note: ** *p* < 0.01.

**Table 12:** Reliability Test of the 14 items.

	Internal Consistency Reliability	Split-Half Reliability	Test-Retest Reliability
Spark	0.857	0.855	0.737
Facilitator	0.906	0.914	0.765
Signal	0.887	0.889	0.788
Overall scale	0.925	0.821	0.860

## Data Availability

The data are not publicly available due to privacy reasons.
